# National Preparedness Month — September 2019

**DOI:** 10.15585/mmwr.mm6835a1

**Published:** 2019-09-06

Every September, CDC, along with partners in government, private and public health, and academia observes National Preparedness Month, a public service reminder of the importance of personal and community preparedness for all events (*1*). This year, CDC’s Center for Preparedness and Response has published a CDC Digital Media Toolkit (https://www.cdc.gov/cpr/npm/npm2019.htm) regarding personal health preparedness, including how to build an emergency supplies kit. In addition to food and water, an emergency supplies kit should include 1) personal needs (supplies necessary to protect physical, mental, and emotional health); 2) an emergency supply of prescription medications and medical supplies; 3) important paperwork including documentation of medical coverage, property ownership, and identity; and 4) backup and alternative power sources for mobile phones and medical devices.

Personal health preparedness is about being able to care for and protect individual and family health in an emergency. Large-scale events, like hurricanes and floods, can cause widespread destruction and long-lasting power outages and strain public health and health care systems. Community preparedness is equally important. This issue of *MMWR* includes a report on participation in community preparedness training in New York City as a model for other U.S. cities (*2*). Additional information on how to prepare your health for emergencies is available at https://www.cdc.gov/prepyourhealth and #PrepYourHealth on Twitter.
